# Advancing Drug Discovery for Neurological Disorders Using iPSC-Derived Neural Organoids

**DOI:** 10.3390/ijms22052659

**Published:** 2021-03-06

**Authors:** Gianluca Costamagna, Giacomo Pietro Comi, Stefania Corti

**Affiliations:** 1 Dino Ferrari Centre, Department of Pathophysiology and Transplantation (DEPT), Neuroscience Section, University of Milan, 20122 Milan, Italy; Gianluca.costamagna@unimi.it (G.C.); giacomo.comi@unimi.it (G.P.C.); 2 IRCCS Foundation Ca’ Granda Ospedale Maggiore Policlinico, Neurology Unit, Via Francesco Sforza 35, 20122 Milan, Italy

**Keywords:** induced pluripotent stem cells (iPSCs), brain organoids, CRISPR-Cas9, drug discovery, disease modeling, neurological diseases, machine learning, single-cell sequencing, bioengineering, organoid imaging

## Abstract

In the last decade, different research groups in the academic setting have developed induced pluripotent stem cell-based protocols to generate three-dimensional, multicellular, neural organoids. Their use to model brain biology, early neural development, and human diseases has provided new insights into the pathophysiology of neuropsychiatric and neurological disorders, including microcephaly, autism, Parkinson’s disease, and Alzheimer’s disease. However, the adoption of organoid technology for large-scale drug screening in the industry has been hampered by challenges with reproducibility, scalability, and translatability to human disease. Potential technical solutions to expand their use in drug discovery pipelines include Clustered Regularly Interspaced Short Palindromic Repeats (CRISPR) to create isogenic models, single-cell RNA sequencing to characterize the model at a cellular level, and machine learning to analyze complex data sets. In addition, high-content imaging, automated liquid handling, and standardized assays represent other valuable tools toward this goal. Though several open issues still hamper the full implementation of the organoid technology outside academia, rapid progress in this field will help to prompt its translation toward large-scale drug screening for neurological disorders.

## 1. Introduction

Organoids are stem cell-derived, three-dimensional (3D) cultures that are artificially generated. Organoids contain different cell types that self-organize through cell-sorting and spatially restricted lineage commitment, similarly to in vivo organs [1]. Different cell types have been used to generate organoids in vitro, including primary cultured cells from human tissues, embryonic stem cells (ESCs), and induced pluripotent stem cells (iPSCs) [2]. iPSC cultures have provided invaluable information for modeling neurological and neuromuscular disorders [3,4]. However, organoids present some advantages over traditional two-dimensional cultures. They exhibit near-physiologic cellular composition, and they can grow extensively in culture while maintaining genomic stability [5,6], making them potentially valuable for high-throughput screenings [7]. Compared with animal models, organoids can reduce experimental complexity and allow the study of human development features that may be difficult to investigate in animal models. Examples of 3D cultures recapitulating human organs in vitro include the peripheral nerve, [8] the spinal cord [9], and the brain [1]. These neural organoids proved to be useful platforms to model neurodevelopmental, neuropsychiatric [10], and neurodegenerative disorders [11], such as microcephaly [12], Miller-Dieker Syndrome [13], and Alzheimer’s disease [14].

**Figure 1:**
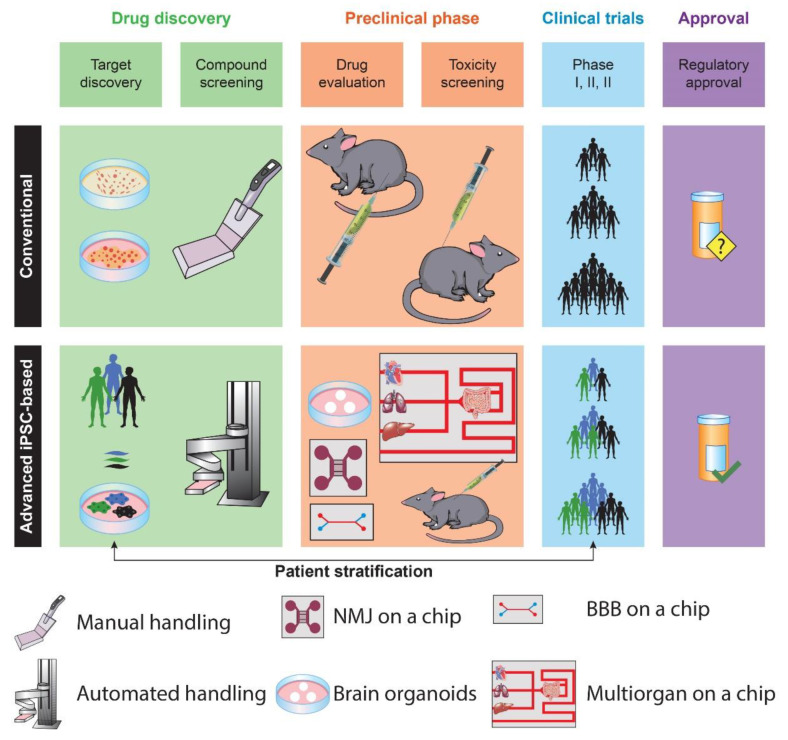
The conventional and iPSC-based drug discovery pipeline.

Animal models represent the gold standard for in vivo modeling in drug development and their widespread adoption across studies has prompted invaluable progress in drug discovery (Figure 1).

The conventional drug discovery pipeline begins with compound screening and target discovery. Then, the efficacy and toxicity of drug candidates are validated in animal models. Based on the results of preclinical studies, clinical trials enable drug testing in humans. Finally, successful drugs are released onto the market (upper row). IPSC-based preclinical studies provide a model with human-relevant genetic backgrounds. In addition, iPSCs enable the generation of 3D cultures with physiologically improved modeling of disease-relevant phenotypes, ameliorating patients’ stratification. These culture systems can be coupled with bioengineering solutions, including organoids, organs (such as neuromuscular junction, NMJ), and a blood-brain barrier (BBB) on-a-chip (lower row), to provide more reliable data for clinical trials and final regulatory approval.

Unfortunately, many therapeutics successful in preclinical model trials fail in late-stage clinical studies due to reproducibility issues between animal models and humans. IPSC-derived organoids permit in vitro and in vivo investigations, representing a relevant innovation to recapitulate the physiological mechanisms of human organs [15]. Particularly, organoid technology could better mimic aspects of human physiology when compared with animal models [1]. Drugs (such as PTC-124 and Ataluren) that were successful in non-neurological animal models were not efficacious in human intestinal organoids modeling cystic fibrosis. These results turned out to be accurate in two-phase clinical studies [16], suggesting that organoids might have the potential to bridge preclinical and clinical trials [17]. However, some challenges still hamper the full application of organoid technology in drug discovery. For instance, the intra-organoid and inter-organoid cellular heterogeneity, the limited scalability, the lack of reproducibility across protocols, and the variable degree of maturity still represent important roadblocks.

Some recent technological advances could overcome these open issues. The advent of CRISPR-Cas9 (Clustered Regularly Interspaced Short Palindromic Repeats/Cas9) gene-editing tools represent a game-changer, opening new avenues in organoid research. Similarly, single-cell RNA (scRNAseq) sequencing could help to delve into the biology of neural organoids at a subcellular level. The adoption of automated systems, machine-learning-based strategies, and high-content imaging (HCI) tools in the generation and analysis of organoids could speed-up the processing of data derived from 3D cultures, potentially enabling the translation of neural organoids from the academic to the industrial setting.

Here, we focus on the early use of neural organoids in the industry, the current challenges, and the possible technical solutions that might support their implementation into drug discovery pipelines.

## 2. Neural Organoids for Drug Discovery in the Industry

The demand for commercial organoids will likely increase in the next few years [18]. In 2019, six companies were already active in the field, whereas at least 19 companies were interested in the business in 2020 [18]. Some of these (e.g., Organome and Hubrecht Organoid Technology) are involved in biobanking, while others are dedicated to manufacturing and commercializing organoids as well as organoid-related technologies. Particularly, different companies show an interest in developing renal, gut, cancer, and neural organoids [18].

Most of the early studies on neural organoids have been conducted in academic settings. However, in the last few years, the interest in neural organoids in the industry has raised, leading to industry-academia collaborations [19].

For instance, Novartis-based and Harvard-based groups collaborated to generate neural organoids to model Zika virus infection [20]. CRISPR-based approaches helped the researchers to knock out the putative entry receptor and to identify viral entry receptors. These organoids displayed an elevated number of viral copies within neural progenitors and exhibited increased cell death, leading to a severe microcephaly-like pattern resembling the infection in vivo [20]. This protocol set the groundwork for a successful collaboration with academia in defining the possible role of the viral attachment factor Tyrosine-protein kinase receptor UFO (AXL) as a potential drug target [19,20].

Stemonix is a company providing iPSC-derived micro-brain organoids for drug screening [21]. Though it is not clear how the process is standardized and reproducible, the company aims to provide neural organoids as a service for disease modeling and drug discovery. Particularly, the focus would be to use this technology as a supplement to the standard drug discovery process. Stemonix has planned to apply organoid technology to discover potential therapeutic approaches for the Rett syndrome [22].

System 1 biosciences is a company dedicated to neurotherapeutic development. They aim to adopt artificial intelligence and robotics-based phenotypic screening on iPSC-derived neural organoids for drug discovery in schizophrenia, autism, and epilepsy [23]. Particularly, they intend to integrate machine learning and high-scale dimensional phenotype analytics to create a comprehensive molecular description of the brain and to reveal paths to new disease-modifying therapies.

A:head bio represents an example of a mixed academia-industry collaboration between a newly formed start-up and the Institute of Molecular Biotechnology (IBMA) in Vienna, Austria. This collaboration aims to develop new therapeutics within central nervous system (CNS) disorders, focusing on Dravet syndrome (a severe epileptic syndrome due to a loss-of-function mutation in the sodium channel SCN1A) [24]. Similarly, in 2006, STEMCELL Technologies and the IBMA signed an agreement to commercialize cerebral organoid culture technologies [25].

Overall, the technology of neural organoids is still in its early stages. While this system may bring some value to the drug discovery pipeline, its use in the industry is limited to recent start-up companies or small teams in larger biotech companies.

## 3. Challenges for the Adoption of Neural Organoids for Drug Discovery

Basic research has benefited from the use of neural organoids, particularly in the field of disease modeling and target identification in the context of neuropsychiatric and neurological disorders [10] (Table 1).

Unfortunately, some obstacles still hamper the implementation of neural factors for large-scale drug screening in the industry. Their degree of heterogeneity and maturity as well their reproducibility and scalability represent some of these hurdles.

### 3.1. Heterogeneity

Sample heterogeneity refers to the inherent variability within biological samples. In the context of large-scale studies—such as in drug discovery research—scientists aim to reduce this variability to reach conclusive and generalizable results. Similar to primary patient samples, both environmental and genetic factors represent potential confounders in neural organoids. The two most important sources of variability include iPSC lines and organoid batch-to-batch variation [42]. The former refers to the variable ability of different iPSC lines to generate relevant architectures and cell types observed in their in vivo counterparts. The latter variability depends on the multitude of reagents and molecules as well as the lack of standardized protocols across experiments used to generate organoids. In addition to this, organoids exhibit a high degree of heterogeneity even within a single batch, reducing the comparability among samples [43,44].

A recent attempt to reduce brain organoid heterogeneity involved the generation of midbrain-like organoids modeling Parkinson’s disease-associated phenotypes using primitive neural stem cells (NSCs). Ha et al. generated NSC-derived organoids in two weeks using either an unspecific or a midbrain-specific medium. These 3D cultures exhibited homogenous cytoarchitectural and transcriptional features, as confirmed with immunochemistry and single-cell analysis [45]. Disease phenotypes were responsive to disease-specific therapies, supporting the potential role of this approach to large-scale disease modeling and drug testing. Similarly, Nickels et al. have recently optimized the generation of midbrain organoids by modifying the timing of maturation, the patterning strategy, and the starting number and type of cells [46]. Particularly, the optimization of timing and the use of a more committed cell line consistently increased the reproducibility of the model, reducing the variation among batches while maintaining the cellular complexity of midbrain organoids.

### 3.2. Scalability

The scalability of the organoid technology represents another challenge. When used for drug screening, the assays are generally performed in 384 or 1536-well plates, leading to adequate control of the study conditions to explore the vast chemical space. However, organoids are typically generated in 96-well plates, growing too big for 1536-well plates, and contain too many cells to be properly fed using 100 μL of media in 384-well plates. The implementation of spinning bioreactors to feed a higher number of organoids has partially overcome this roadblock [47,48], but limitations regarding drug screening remain. The adoption of engineering solutions for micro-fluidic and milli-fluidic systems, automated liquid handling (see “Automation and high-throughput screening”), and robotic manipulations may overcome this issue, enhancing scalability for drug screening approaches.

### 3.3. Reproducibility

Available protocols typically use Matrigel, which is the gelatinous protein mixture secreted by mouse sarcoma cells, as an extracellular matrix (ECM)-like structure to support proper organoid formation [1]. Physiologically, different cell types contribute to ECM formation and maintenance, including microglia and vascular endothelial cells, which may lack in current neural organoids. Though Matrigel is extensively adopted, its composition is generic and largely uncharacterized, increasing variability and lacking the physiologically-relevant biochemical signals driving organoid generation.

Some recent developments have tried to recreate a more physiological microenvironment for organoids and to increase their reproducibility. For instance, the use of ECM derived from fetal porcine brains combined with silk scaffolding improves the functional maturation of neural populations, better supporting their long-term growth [49]. In another case, Lancaster et al. used polylactic-co-glycolic acid (PLGA) molecules, which are synthetic biocompatible microfilaments, as a basement membrane-like structure for self-patterning organoids, generating the microfilament-engineered cerebral organoids (enCORs) [50]. This scaffolding supported the cytoarchitectural organization of enCORs, improving radial organization and highlighting the role of tailored structures in improving organoid development. Different studies have applied bioprinting technologies to support regulated, cellular patterning at specific locations within the cultures, providing a spatial control of cell development [51,52], thus, increasing the reproducibility of organoids during differentiation.

### 3.4. Maturity

The degree of maturity may limit the potential of neural organoids to model adult-onset disease in vitro. Organoids may lack neuro-endothelial-glial-immune inter-lineage signaling if compared with their in vivo counterparts [42]. In addition to this, spontaneous vascularization lacks neural organoids, leading to aberrant maturation. As a result, late-stage organoids have inadequate nutrient delivery, accumulate waste, and develop central necrotic cores. This process may have two consequences. First, the hypoxia-induced release of necrosis-associated cytokines may lead to paracrine effects disrupting the function of neighboring vital cells, ultimately, impairing the sample analysis. Second, the limited diffusion of nutrients may account for the lack of stereotypical anatomy in neural organoids [53].

Integrating a functional vascular network within neural organoids is crucial to accurately model later stages of brain maturity. Different groups have adopted various methods toward this goal. Some laboratories have generated vascularized neural organoids using patients’ iPSC-derived endothelial cells [54] or by in-vivo transplantation in mice [55], creating the chimeras [56]. Ham et al. treated early brain organoids with vascular endothelial growth factor (VEGF) and WNT7b to favor the differentiation of endothelial and pericyte-like cells without altering neuronal populations [57]. This protocol led to the generation of open-circle vascular structures containing CD31-positive cells and to the modulation of gene networks associated with brain embryogenesis in neuronal populations, suggesting a cross-talk between neurons and endothelial cells [57]. Wörsdörfer et al. applied a co-culture method using mesodermal progenitors integrated into brain organoid cultures, generating a vessel network with a blood vessel-like ultrastructure after eight weeks in vitro [58].

Vascularized organoids display a blood-brain barrier (BBB)-like characteristics [59]—such as expression of tight junctions, nutrient transporters, and trans-endothelial electrical resistance—offering invaluable advancements for drug discovery pipelines, as the BBB represents a key limiting factor for drugs targeting the CNS. Other organoid-based studies showed the possibility of generating complex BBB models containing various cell types, including pericytes, astrocytes, microglia, and oligodendrocytes, which are susceptible to functional assessment testing and potentially valuable for toxicity testing and drug discovery [60,61].

Other approaches to bypass diffusion limitations include the generation of sliced neocortical organoids (SNs), allowing improved oxygenation into organoid cores and promoting long-term neurogenesis, sustained progenitor expansion, and separation of cortical layers [62]. Similarly, Giandomenico et al. have cultured sliced, cortical organoids at the air/liquid interface [63]. These organoids exhibited improved neuronal survival compared with whole organoids as well as mature morphological and functional features, such as long-range axons. These axons could elicit coordinated muscle contractions in co-cultured mouse spinal cord–muscle explants.

The time needed to reach organoid maturity may represent another roadblock to widespread adoption. Currently, though novel protocols are available [47,64], most cerebral organoids might require months [1] to reach full development. Innovative strategies to speed up growth or processing [65] might facilitate their use for drug discovery.

In the following section, we will discuss the possible technical solutions to improve the implementation of neural organoids in drug discovery. These include CRISPR-Cas-9 to generate isogenic models and facilitate disease modeling of genetic disorders, advanced imaging, and single-cell sequencing approaches to characterize the diseased phenotypes more accurately, automated systems to accelerate the production, and machine learning to speed up the analysis of these complex data sets. Engineering solutions such as automated liquid handling and optimized plate systems represent other important approaches as well.

## 4. Overcoming Challenges with Possible Technical Solutions

### 4.1. CRISPR

**Figure 2:**
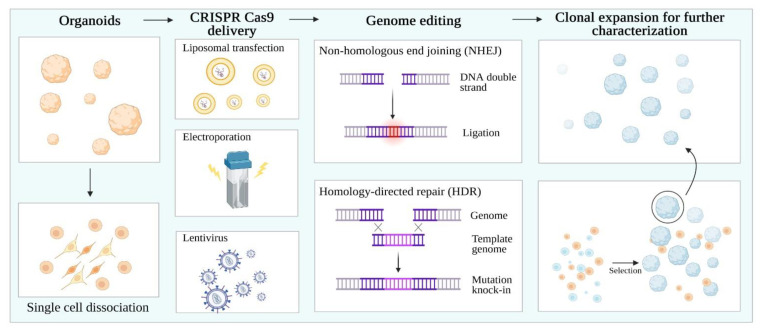
Genome editing of organoids using Clustered Regularly Interspaced Short Palindromic RepeatsCRISPR-Cas9technology.

CRISPR/Cas 9 (Clustered Regularly Interspaced Short Palindromic Repeats/Cas9) technology has evolved the field of functional genomics, enabling the generation of isogenic iPSC lines at an unprecedented speed, precision, and flexibility. This has enabled the interrogation of the role of a specific mutation in the same genetic background. The CRISPR/Cas9 system is based on a guide RNA to target the nuclease Cas9 to a specific genome locus, causing a double-strand break and activating DNA repair mechanisms, such as homology-directed repair (HDR) and non-homologous end joining (NHEJ) repair pathways [66] (Figure 2).

Methods harnessing the CRISPR-Cas9 technology in organoids involve dissociation into single cells. Then, liposomal transfection, electroporation, and lentiviruses can be used to genetically engineer organoids. Non-homologous end joining (NHEJ) facilitates gene knock-out, while homology-directed repair (HDR) enables gene knock-in. Finally, single organoid clones can be selected and expanded to obtain isogenic cell populations.

NHEJ represents the gold standard to create gene knockouts by CRISPR. Cas cleavage followed by NHEJ-mediated repair at the break site is repeated until an error—usually a small indel—occurs. As shown in seminal studies on mice, these indels are valuable to introduce frameshift mutations for the generation of gene knockouts [67,68].

Considering in vitro disease modeling, CRISPR-Cas9 is typically performed on neural organoid founder cells, such as ESCs and iPSCs [13,69,70]. Then, neural organoids can be grown from selected cells carrying the mutation, with the advantage that all forming cells contain the desired genetic change. Alternative methods to introduce CRISPR/Cas9-mediated genetic changes in growing organoids exist. Plasmid vectors containing Cas9 and one or more guide RNAs [71] can be electroporated in early organoids, which are known as embryoid bodies, inducing loss-of-function mutations, as seen in organoid models of CNS tumors [34]. Another example includes the combination of CRISPR-Cas9-directed tumor suppressor (TP53) knock-out with oncogene Kirsten rat sarcoma (*KRAS*) knock-in in a four-month-old neural organoid-based glioblastoma model [72]. In this case, the proliferative potential of several glioblastoma-like cells is sufficient to provide a persistent genetic modification within the organoids.

Though CRISPR has not been used for high-throughput screening using neural organoids yet, this technology has provided new tools for disease modeling. Some examples include neuropsychiatric disorders, such as Miller-Dieker Syndrome [13,73], autism [74], macrocephaly [70], and neurological diseases, such as tumors, including glioblastoma [72] and retinoblastoma [69], viral infections, Zika Virus (ZV) [19], and neurodegenerative disorders, including Alzheimer’s disease and frontotemporal dementia [14,75]. Particularly, gene editing on neural organoids infected with ZV has allowed the study of ZV entry receptors. This system relied on a doxycycline-inducible Cas9 and the introduction of a single gRNA via lentivirus, promoting the efficient knock-out of the AXL receptor, demonstrating that this receptor was unnecessary to drive ZV infection [19].

CRISPR-Cas technology applied to model neuro-ophthalmic conditions has driven an important advancement in the field. This technology used in patient-derived iPSCs [76] has paved the way to a recent CRISPR-based clinical trial in patients with Leber congenital amaurosis (NCT03872479) and congenital retinal dystrophy leading to severe vision loss at an early age. A construct containing an adenovirus vector AAV5 with dual single-guide RNA (sgRNA) mediated the gene correction of the IVS26 cryptic-splice mutation in Centrosomal Protein 290 (CEP 290) in the retina [77]. Correction of retinitis pigmentosa GTPase regulator (RPGR) mutations in iPSCs followed by the generation of retinal organoids proved to be a valuable approach for testing new potential therapies in retinitis pigmentosa [78]. In parallel to therapy testing, neuroophthalmological disease modeling profited from CRISPR-Cas as well. The introduction of the E50K mutation in the optineurin (OPTN) gene enabled the modeling of glaucoma in retinal organoids [79] and the knockout of RB1 demonstrated its crucial role in retinal development [80].

Similarly, Magil et al. have recently applied a CRISPR-Cas interference-based approach in brain cancer organoids [81]. Particularly, they generated meningioma cells expressing a specific Cas9 from patients’ tumor samples and co-cultured them with brain organoids. Then, they repressed two key genes involved in meningioma proliferation (CHD2 and PTPRZ1) by transducing cells with lentiviruses harboring the single guide RNA (sgRNA) targeting the genes. This CRISPR-induced repression attenuated tumor cell proliferation and was valuable in defining the potential role of chromodomain helicase DNA binding protein 2 (*CHD2*) and Protein Tyrosine Phosphatase Receptor Type Z1 (*PTPRZ1*) as novel targets for molecular therapy to treat meningioma patients.

Overall, these findings suggest the valuable role of CRISPR in disease modeling and therapy testing using disease neural organoids and their isogenic counterparts.

### 4.2. Automation and High-Throughput Screening

Compared with organoid-based studies for cancer [82,83], automation in the generation and analysis of neural organoids is still in the early stages. Established protocols tend to lack a focus on scalable, homogenous organoids with a predictable morphology, cellular composition, and organization. Extensive manual handling includes complicated matrix embedding steps, rendering organoids painstaking to culture as well as time-consuming and resource-consuming as limiting the scale-up [84]. In addition, common analysis methods (e.g., tissue sectioning, immunohistochemical analysis, RNA sequence) do not perfectly fit high-throughput screening (HTS) requirements.

As already demonstrated using kidney organoids, liquid-handling robots can perform all steps of cell culturing, including plating, differentiation, and fixation [85]. The use of robots coupled with software trained to recognize organoids in microwells and to perform quantitative imaging analysis that enabled organoid phenotyping, providing an attractive starting point for high-quality HTS and possible application as a therapeutic screening tool. Recently, Renner et al. have used a similar system to generate a fully-automated HTS workflow to generate and analyze midbrain organoids [86]. The authors used a liquid-handling robot that could perform different steps, such as seeding, maintenance, fixation, whole-mount staining, and clearing in a fully scalable automated fashion in standard 96-well-plates. The resulting midbrain organoids showed small intra-batch and inter-batch variability in size distribution, morphology, and cellular composition as well as comparable morphological features compared with other studies [86]. Further characterization using RNA sequencing and quantitative whole mount HCI confirmed the reproducibility of these organoids.

Though preliminary, these automated methods from seeding to analysis in standard plates could represent a feasible scale-up and implementation into existing, screening facilities.

### 4.3. Imaging

Morphological analysis and microscopic dissection of human organoids are invaluable to investigate their cytoarchitecture. Fluorescence microscopy theoretically enables the study of intact organoids with high spatiotemporal resolution (nanometer and millisecond), providing a potential tool for spatiotemporal reconstructions known as 4D imaging. Unfortunately, different roadblocks have hampered their use in this context. Both traditional fluorescence microscopy and computational methods for 3D deconvolution are not ideal for studying organoids. In particular, the first struggle to sample 3D structures thicker than 10 micrometers, whereas the second faces challenges in properly deconvolving complex, multilayered architectures. To address these limitations, early organoid-based studies have implemented thin-sectioning methods, such as cryo-sectioning to obtain suitable samples for microscopy [1]. Though useful, these techniques are labor-intensive, impair the living tissue, and dismantle the 3D cytoarchitecture. Recently, various new approaches have allowed a quicker and a more traumatic observation of organoids.

The most common method for in vivo optical section is laser point scanning, as applied in confocal and two-photon microscopy [87,88]. Laser point scanning is based on a small laser point moved sequentially across a sample to different 3D positions, while an image is created by assigning the signal detected at each point with its position in 3D space. 3D microscopy is available in various commercial HCI systems. While many different confocal implementations exist, all these approaches share the same principle. Out-of-focus light is excluded from the microscope’s camera by using a pinhole. Several different flavors of confocal microscopy are commonly employed, from point scanning systems to spinning disc microscopy, compromising between Z resolution (axial) and speed. While point scanning devices offer sharper and deeper images in the sample, they are slower than spinning disc microscopes. Despite enabling 4D observations, they cause phototoxicity due to out-of-focus light, reducing their applicability to study living organoids. Multi-photon microscopy represents a noninvasive optical sectioning method to explore thick tissue samples and fine cellular details with high depth and low phototoxicity [89], but its commercial use is still limited.

Light-sheet fluorescence microscopy (LSFM) has emerged as a particularly fast and gentle technology, enabling both high-resolution imaging and live-cell microscopy. Unlike point-scanning, LSFM illuminates the regions of the sample being actively imaged by the device. This reduces laser power and phototoxicity while increasing the image dimension and rate of acquisition [90]. Low phototoxicity permits the imaging of a sample for hours or days, with clear advantages in terms of temporal analysis. Traditional LSFM requires specific sample mounting, making it unsuitable for automation or multi-well plates. Recent advances might enable 4D imaging in multi-well plates providing quicker analysis. The use of single objective light-sheet techniques [91,92,93] (instead of multiple objective lenses) has enabled low-resolution imaging of transparent multi-well plates. In parallel, improvements in optics [94] have increased the resolution of this technique. The commercialization of these systems will likely enable 4D imaging using available automation technologies.

#### Pitfalls in Organoid Imaging

Elevated scattering of light represents a common challenge in imaging complex biological tissues, including organoids. This phenomenon derives from the high structural complexity of these 3D cultures, particularly due to deposits of proteins, carbohydrates, and lipids [93]. Recent potential solutions to reduce tissue scattering include the use of adaptive optics compensating for the specific scattering of each sample, as shown in studies with drosophila and zebrafish [95,96]. However, the diffusion of these approaches is still limited and not widely commercialized.

Fixed organoids can be chemically modified to render them transparent and reduce light scattering. Early tissue clearing aims to reduce the scattering of tissue by replacing water with oils matching the refractive index of lipids’ membranes [97]. Different methods have been used, including solvent denaturation, hyperhydration, and tissue embedding [98]. The most common approach is clear lipid-exchanged acrylamide-hybridized rigid imaging/immunostaining/in situ hybridization-compatible tissue-hydrogel (CLARITY) [99]. This system is based on porous hydrogel tissue embedding and electrophoresis to remove lipids while preserving the structure and native biomolecules such as protein and nucleic acids. Its application on neural organoids has already enabled 3D imaging of whole cerebral organoids using LSFM [100]. However, none of the previous tools has seen widespread use in neural organoid-based studies yet. Future high-content screening platforms should focus on the compatibility of the clearing methods with fluorophores, dyes, and antibodies used for the characterization of organoids.

Organoids can be challenging for dyes and antibodies to diffuse through, limiting the application of traditional methods. Even well diffusive dyes such as 4′,6-diamidino-2-phenylindole (DAPI) may encounter difficulties in staining DNA in the cells toward the center of an organoid. Some solutions include the use of genetically encoded fluorescent proteins—especially red-shifted labels—coupled with tissue clearing methods. Fluorescence In Situ Hybridization (FISH) probes and antibodies seem to diffuse particularly well through cleared tissues, enabling the detection of proteins and nucleic acids [89,101]. However, many fluorescent dyes have scarce compatibility with clearing techniques [102,103], which is an aspect to properly evaluate before staining.

Prospectively, organoid imaging techniques might include highly multiplexed approaches, such as transcriptome mapping [104] and proteome imaging [105,106,107], using small nucleic acid probes and antibodies diffusing through cleared tissue. Transcriptome technologies based on in situ hybridization fluorescence with confocal microscopy can image up to 10,000 genes in single cells with high accuracy and resolution [108], enabling both morphological and transcriptomic phenotyping. Proteome imaging techniques achieve comprehensive profiling of intracellular protein maps with a quantitative description of compartmentalized intracellular proteins [106]. These maps help to identify cellular states and to define the intracellular organization between single cells in different cell cycle states and therapeutic contexts.

Altogether, these findings show that, despite some current technical challenges related to organoid imaging, recent progress in high-resolution imaging coupled with multiplexed approaches will likely enable valuable biological insights for drug discovery.

### 4.4. Single-Cell RNA Sequencing

Scientists have used different emerging techniques to delve into the biology of neural organoids, including scRNAseq. Historically, researchers have performed gene expression studies on “bulk” tissue samples composed of many cells of different types and functions. This approach has yielded valuable results in comparing general characteristics of tissues—such as pre-treatment and post-treatment conditions—though presenting a low-resolution for elucidating cellular composition within a tissue [109]. Advances in sgRNAseq have enabled us to profile gene expression in individual cells on a large scale, helping to investigate the heterogeneity across cell types, including those in complex neural organoids [110].

Although scRNAseq has not reached a full stride in neural organoid technology for drug discovery—differently from the preliminary results on cancer organoids [111], including glioblastoma [112,113,114]—this approach has opened up new possibilities in exploring neurodevelopment and cell phenotyping [42]. For instance, Quadrato et al. have highlighted a considerable variability of cellular populations within organoids [43]. Using a high-throughput droplet-based microfluidic approach known as Drop-seq [115], they analyzed 80,000 cells from 31 organoids grown over an extended period, providing the first molecular map of the variety of cells generated in neural organoids. The authors detected a large diversity of cell classes from different regions of the brain and retina, including glial cells and mature neurons. In addition, this cellular composition diversified over time, showing a progressive level of maturity.

**Figure 3:**
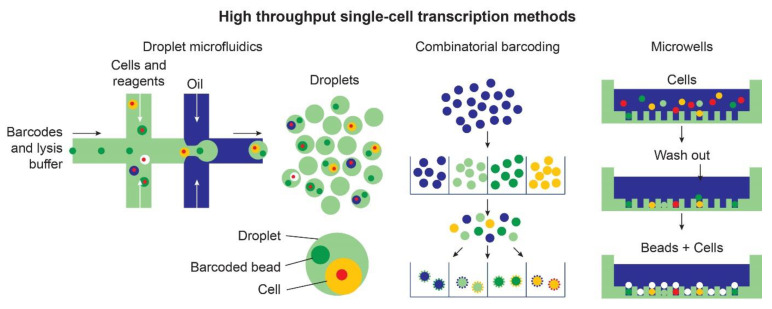
Single-cell RNA sequencing approaches to investigate cell heterogeneity in human organoids.

The use of RNAseq has enabled researchers to bypass some of this variability by filtering out cells of no interest, increasing the granularity of the analysis (Figure 3).

Different high-throughput methods based on droplets microfluidic, combinatorial barcoding, and micro-well technologies have expanded the number of cells that can be investigated in each experiment. These approaches enable the quantitative comparisons of differential cell states within organoids.

However, the most frequently applied systems become labor-intensive and cost-prohibitive when processing more than 20 samples at a time [98]. Emerging “cell-hashing” approaches, including multiplexing for single-cell RNA sequencing (MULTI-seq) and Cellular Indexing of Transcriptomes and Epitopes by Sequencing (CITE-seq), might be more efficient in multiplexing single cells for scaled projects [116,117]. The MULTI-seq approach refers to the use of lipid-modified and cholesterol-modified oligonucleotides as sample barcodes in live cells and nuclei, regardless of species and genetic background, improving scRNA-seq data quality. By integrating this method with automated liquid handling, MULTI-seq might serve as a valuable technology to perform “screen-by-sequencing” (such as the DRUG-seq method [118]) in multicellular systems, including organoids [116]. The CITE-seq is based on oligonucleotide-tagged antibodies targeting cell surface proteins that uniquely label different experimental samples. This barcoded antibody signal—known as “cell hashing”—serves as a reference for assigning each multiplexed cell to its original sample. With this tool, different cellular samples can be labeled, pooled, and finally run simultaneously in a single-droplet scRNA-seq. This technology might enable a consistent sample multiplexing, precise multiplet identification, and a cost reduction of commercial scRNA-seq platforms [117].

Some limitations still hamper the widespread application of this method for organoids. Though rapidly decreasing, the cost of sequencing is still considerable. The challenges related to cell dissociation of organoids (e.g., dissociation of mature neurons from older organoids and batch effects introduced by dissociation [119]) still exist.

Overall, these findings suggest that the use of scRNA-seq for drug discovery in neural organoids is still in its formative years. However, its future application might provide valuable advancements in the field.

### 4.5. Machine Learning

Machine learning refers to a branch of artificial intelligence regarding computers that can improve their performance through experience, i.e., training. It is a rapidly growing technical field lying at the intersection between computer science and statistics. In cell biology, machine learning teaches computers to recognize phenotypes. Particularly, machine learning aims to learn processing rules from examples rather than relying on manual adjustments of parameters or pre-defined processing steps [120]. In this way, computational data analysis reduces the workload for the experimentalists and permit objectivity and consistency in the processing of large datasets. Machine learning typically proceeds in two phases. In the training phase, data samples are used to build or improve a computer system by learning from inherent structures and relationships within this data. In the second phase, this computer system is used to infer the properties of the data samples. The ultimate goal is to generalize from a few training examples to make predictions on larger data sets not observed during training [121].

#### 4.5.1. Machine Learning in Drug Discovery Using Neural Organoids

Machine learning has proved to be useful in various stages of drug discovery, including analysis of digital pathology data in clinical trials, identification of prognostic biomarkers, and target validation [122]. Different machine learning methods, including the support vector machine [123], K nearest neighbors [124], naïve Bayes [125], random forest [126], and many others [120] have provided valuable insights in this context. In addition to these approaches, recent developments in deep neural networks [127] have catalyzed the interest and have sparked further research in the field. Machine learning approaches in the pharmaceutical industry are commonly used for virtual screening of compounds, performing low to medium-throughput screening [128]. Similar machine learning tools have been used in toxicological research [129].

Machine learning methods for drug discovery in neural organoids are still in their infancy. Most of the studies using organoids have focused on the phenotypical characterization of the model using machine learning tools. A study based on iPSC-derived cortical organoids grown over several months documented a similarity between the electrophysiological activity pattern of cortical organoids and human preterm neonatal electroencephalography using supervised machine learning [130]. In another study, iPSC-derived retinal organoids adopted a deep-learning computer algorithm based on bright-field imaging to recognize and predict retinal differentiation [131]. Particularly, they applied a transfer learning approach using convolutional neural network (CNN) algorithms that are loosely modeled on the network of a human brain agglomerated into multiple functional layers [87]. CNN processing consisted of two phases: a pretraining on the ImageNet classification Dataset [132] and the actual prediction of retinal differentiation in organoids, showing the ability of CNN-based approaches to classify stem-cell-derived tissues in vitro.

Regarding machine learning for drug discovery in neural organoids, the available literature is scant. A study by Monzel et al. developed a machine learning-based HCI analysis using Parkinson’s disease (PD) human midbrain organoids (hMO) exposed to toxic compounds. The researchers analyzed in vitro toxicity mediated by 6-hydroxydopamine (6-OHDA), a toxic molecule specifically targeting dopaminergic neurons, using HCI coupled with random forest classification. This approach discriminated between controls and treated organoids, predicting neurotoxin-induced cellular perturbations with a 75% accuracy [133].

Despite a widespread application of machine learning in drug discovery with organoid platforms still lacking, rapid progress in this field might advance their use in the future.

#### 4.5.2. Future Perspectives on Machine Learning Applied to Organoid Technology

Culturing cerebral organoids has enabled high-throughput screening to test genetic and chemical modifications on cellular phenotypes [2]. Quantification of these phenotypes has historically been a laborious process, involving painstaking manual work. However, recent advances in microscopy and single-cell sequencing coupled with novel computing systems and data processing might support the description of phenotypes at an unprecedented speed.

High-content microscopy is fundamental to provide a characterization of cell components. In many instances, scientists rely on manual measurements of organoid phenotypes [134]. Some workflows permit a more rapid nucleus identification, neurite segmentation, and nucleus-to-cytoplasmic ratio calculation, limiting time-consuming work [135,136]. Recently, the use of CNN on microscopy imaging has improved cell phenotyping under various conditions [137,138], increasing imaging resolution [139], synapse quantification [140], and neurite measurements [141].

ScRNA-seq platforms can characterize cell types and states and they have offered new insights into the phenotyping of cellular systems (see single-cell RNA sequencing). However, as the scale of single-cell studies continues to grow, the increasing number of cells and the batch effect represent computational challenges. Emerging deep learning methods might represent a valuable tool to unfold complex cellular heterogeneous samples—such as organoids—using scRNA-seq [142,143]. Deep-learning refers to representation-learning methods with multiple layers of representation derived from the combination of single modules. Each module transforms the representation at one level (starting with the raw data) into a representation at a higher level [144]. The key aspect of this approach is that these layers of features are not human-driven but learn from data using a general-purpose learning process. Some examples include the Deep Embedding for Single-cell Clustering (DESC) method and scDeepCluster tool. The DESC approach proposed by Li and colleagues clusters scRNA-seq data through iterative clustering and self-learning [142], removing complex batch effects among cellular samples while preserving biological variations. ScDeepCluster refers to a clustering method that simultaneously learns future representation and clustering through modeling of scRNA-seq data generation [143], providing a valuable algorithm for large-scale scRNA-seq data. Other computational models that might help to analyze the amount of data from scRNA-seq of heterogeneous cellular samples include models of RNA splicing dynamics, enabling the study of the cell state and development [145,146,147].

Altogether, these findings suggest that high-content microscopy and scRNA-seq methods coupled with advances in computational tools might provide more reliable and cost-effective analysis of heterogeneous cellular models, including neural organoids.

## 5. Conclusions

The organoid technology is potentially revolutionary and might bridge the gap between preclinical and clinical trials, enabling more reliable testing models for precision medicine and drug discovery. So far, organoid-based research has been mainly conducted in academic environments, with only a few companies actively investing in the technology for scalable manufacturing. The target customers of these development companies include primarily academic researchers and larger pharmaceutical companies. Some of them provide media, reagents, and products for organoid development, while only a handful of them are commercializing whole organoids as products. However, many companies have pending patents [18] for organoid development and culturing, aiming to ramp up the production when the technology will be ripe for commercialization on a larger scale.

The integration of innovative bioengineering solutions could accelerate organoid manufacturing. The application of CRISPR-Cas technologies will help to provide more reliable disease models. The implementation of HCI and scRNA sequencing coupled with machine learning tools might enable the processing of larger datasets more rapidly. In addition, the use of automated systems will help to accelerate the generation of organoids. Microfluidic organoid-on-a-chip platforms [148] could provide an accurate system for consistent organoid perfusion, mimicking a more physiological nutrient supply. The confluence of different disciplines, including tissue engineering, artificial intelligence, and automation, will likely help to achieve a better reproducibility, scalability, and efficiency of the organoid model and to prompt its translation toward large-scale studies for drug discovery of neurological disorders.

## Figures and Tables

**Table 1:** Selected studies using neural organoids to model neurological diseases.

Disease Group	Diseases Modeled	Organoid Type	Intervention	Disease Phenotype	References
Neurodevelopmental	Primary microcephaly	Undirected	Electroporation-mediated *CDK5RAP2* overexpression and knockdown (with shRNAs)	Smaller organoids; reduced number of radial glial stem cells; premature neural differentiation; rescue by shRNA for *CDK5RAP2*	[12]
	ASPM-related microcephaly	Forebrain	Electroporation-mediated knockdown of *Aspm*	Poorly organized neuroepithelium, reduced number of radial glial cells, and defective cortical layer lamination	[26]
	Seckel syndrome	Undirected	Small molecule-mediated and siRNA-mediated inhibition of the cilium disassembly complex	Smaller size and premature neuronal differentiation; increased number and length of cilium; retarded cilium disassembly.	[27]
	Periventricular heterotopia	Undirected	Induced pluripotent stem cell (iPSC)-derived organoids from patients carrying cadherin receptor-ligand *DCHS1* and *FAT4* gene mutations	Morphological alterations, defective migration, axonal guidance, and patterning of neural progenitors.	[28]
	Neonatal hypoxic injury	Forebrain	Low oxygen exposure controlled with a gas chamber and fiber-optic microsensors	Impairment of intermediate progenitors in the subventricular zone; altered activation of cellular stress response pathways and early neuronal differentiation; phenotype rescue by stress response inhibitor (ISRIB)	[29]
	Down syndrome	Ventral forebrain/chimeric mice	iPSC-derived organoids from patients	Increased expression of GABAergic interneurons; shRNA-knockdown of *OLIG2* rescues phenotype in vitro and ameliorates behavioral deficits in mice	[30]
Neurodegenerative	Alzheimer disease	Forebrain	Herpes Simplex virus-1 (HSV-1) -infected organoids derived from human induced neural stem cells seeded into a biomaterial-based scaffold.	Aβ plaque formation, neuronal loss, reactive gliosis, neuroinflammation, and diminished neural network functionality	[31]
	Parkinson disease (PD)	Midbrain	iPSC-derived organoids from patients carrying the *LRRK2-GS019S* mutation data	Recapitulation of PD-specific pathological signatures, including increased α-synuclein. Upregulation of TXNIP favored disease phenotype.	[32]
	Creutzfeld-jakob disease (CJD)	Undirected	Cellular inoculation within the organoids with human brain homogenates from sporadic CJD subtypes	Reduced neuronal metabolism, deposition of insoluble protease-resistant (PrP) aggregates with seeding activity, over activation of astrocytes	[33]
Brain Tumors	Glioblastoma	Forebrain	Electroporation-mediated plasmid infection leading to overexpression of *MYC* and inhibition of tumor suppressor genes	Glioma-like cells with poor differentiation, increased cell proliferation and disrupted cytoarchitecture, downregulation of PI3K-AK and RAS pathways, tumor invasion upon animal transplantation, partial rescue in glioma-like organoids using EGFR-inhibitors	[34]
Infectious	Herpes Simplex virus	Forebrain	iPSC-derived organoids infected with HSV-1	HSV-1 transportation from the periphery to the central layers of the organoids, spontaneous reactivation of viral latent infection	[35]
	Cytomegalovirus (CMV)	Undirected	CMV-infected hiPSCs differentiated into neural organoids	Impaired cell proliferation, tissue degeneration with necrosis, vacuoles, and cysts, disrupted cortical lamination	[36]
	Zikavirus (ZIKV)	Forebrain	Treatment with RNA interfering (RNAi) enhancer (enoxacin)	Microcephaly-like phenotype, production of small interfering RNAs in neural progenitors upon infection, viral clearance using enoxacin	[37]
Psychiatric	Schizophrenia	Forebrain	iPSC and embryonic stem cell (ESC)-derived organoids from patients. Chemically mediated neuronal (n)FGFR modulation.	Abnormal neuronal migration, particularly towards the cortex, decreased intraneuronal intracortical connectivity. Both loss of function and hyperactivity of neuronal (n)FGFR1 affects cortical neurodevelopment.	[38]
Prenatal toxin exposure	Nicotine	Undirected	Organoids cultured with microfluidic devices. 3D cultures exposed to a physiologically relevant concentration of nicotine.	Premature neuronal differentiation, disrupted brain regional organization, abnormal cortical development, and neuronal outgrowth,	[39]
	Methamphetamine	Undirected	10-month-old ESC-derived organoids treated with methamphetamine for a week	Increased expression of astrocyte-specific gene networks related to inflammations. Upregulation of genes involved in complement activation, apoptosis, and immune response.	[40]
	Cannabis	Undirected	ESC-derived organoids grown in a microfluidic platform and perfused for 27 days with Δ-9-tetrahydrocannabinol (THC)	Reduced neuronal maturation and spontaneous firing, downregulation of cannabinoid receptor type 1 (CB1) receptors, and impaired neurite outgrowth.	[41]
